# Supplementary material for: Multifunctional Oxidized Dextran as a Matrix for Stabilization of Octahedral Molybdenum and Tungsten Iodide Clusters in Aqueous Media
Source: Int J Mol Sci. 2023 Jun 11;24(12):10010. doi: 10.3390/ijms241210010 (PMC10297999; doi:10.3390/ijms241210010)
Supplement: Supplementary file 1 [file ijms-24-10010-s001.zip › ijms-2443255-supplementary.pdf]

Supporting Information

Multifunctional oxidized dextran as matrix for stabilization of octahedral molybdenum and tungsten iodide clusters in aqueous media

Ekaterina V. Pronina [1], Yuri A. Vorotnikov [1,*], Tatiana N. Pozmogova [1], Alphiya R. Tsygankova [1], Kaplan Kirakci [2], Kamil Lang [2], Michael A. Shestopalov [1]

Table S1. Chemical groups content and molecular weights of initial and oxidized dextrans [1].

Sample	Carbonyl groups, mmol·g^{-1}	Acidic groups, mmol·g^{-1}	M_w, kDa
Dex6	-	-	6.2
NaOxDex6(1)	0.6	1.4	3.8
NaOxDex6(2)	0.6	2.3	3.9
Dex60	-	-	60.2
NaOxDex60(1)	1.0	1.0	14.0
NaOxDex60(2)	1.3	1.5	4.5
NaOxDex60(2)-red	0.8	1.5	4.5

1. Pronina, E.V.; Vorotnikov, Y.A.; Pozmogova, T.N.; Solovieva, A.O.; Miroshnichenko, S.M.; Plyusnin, P.E.; Pishchur, D.P.; Eltsov, I.V.; Edeleva, M.V.; Shestopalov, M.A.; et al. No Catalyst Added Hydrogen Peroxide Oxidation of Dextran: An Environmentally Friendly Route to Multifunctional Polymers. *ACS Sustain. Chem. Eng.* **2020**, *8*, 5371-5379, doi:10.1021/acssuschemeng.0c01030.

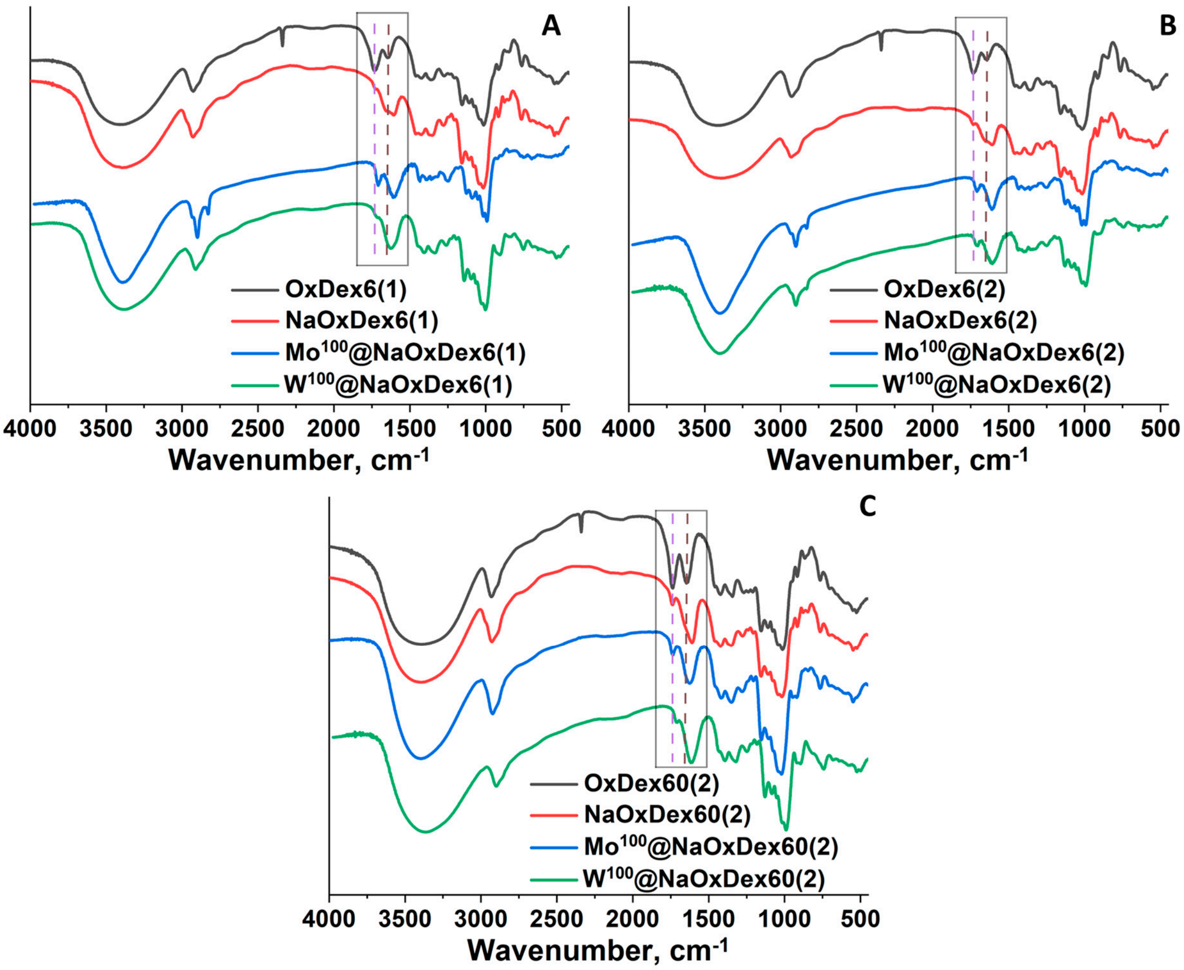

Figure S1. FTIR spectra of OxDexQ(n), NaOxDexQ(n), Mo^{100}@NaOxDexQ(n), and W^{100}@NaOxDexQ(n) (A – Q = 6, n = 1; B – Q = 6, n = 2; C – Q = 60, n = 2).

Table S2. {M_6I_8} content in materials (μmol·g^{-1}) according to ICP-AES

x	10	50	100
		**M = Mo**	
Dex6	-	-	28
NaOxDe6(1)	33	122	139
NaOxDe6(2)	31	127	144
Dex60	-	-	54
NaOxDe60(1)	31	97	115
NaOxDe60(2)	30	118	130
		**M = W**	
Dex6	-	-	48
NaOxDe6(1)	11	51	82
NaOxDe6(2)	13	51	87
Dex60	-	-	59
NaOxDe60(1)	20	49	125
NaOxDe60(2)	16	58	119

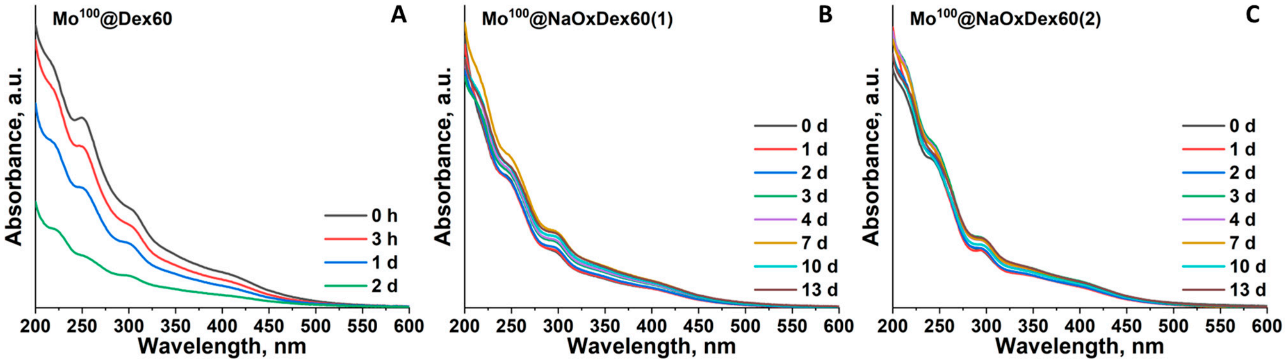

Figure S2. UV-vis spectra of Mo^{100}@Dex60 (A), Mo^{100}@NaOxDex60(1) (B), and Mo^{100}@NaOxDex60(2) in water over time.

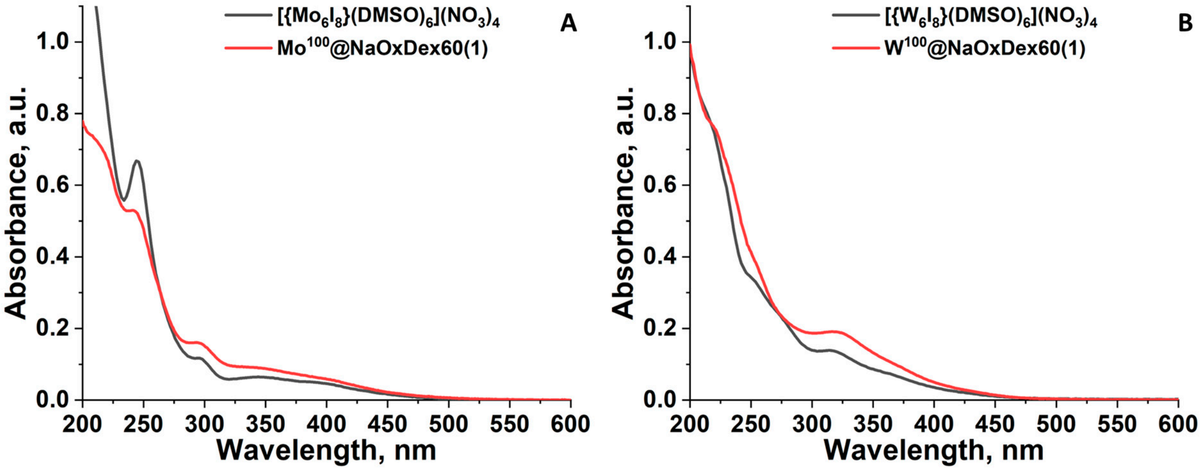

Figure S3. UV-vis spectra of M^{100}@NaOxDex60(1), M = Mo (A) and W (B) in water, in comparison with corresponding initial clusters.

Table S3. Formulation of culture medium DMEM.

Component	Concentration, mg/L	Component	Concentration, mg/L
**Inorganic salts**		**Amino acids**	
Calcium Chloride · $2H_2O$	264.92	L-Arginine · HCl	84.00
Ferric Nitrate · $9H_2O$	0.10	L-Cystine · 2HCl	62.58
Magnesium Sulfate (Anhydr.)	97.67	L-Glutamine	584.00
Potassium Chloride	400.00	Glycine	30.00
Sodium Chloride	6400.00	L-Histidine · HCl · H_2O	42.00
Sodium Phosphate, Monobasic (Anhydr.)	108.69	L-Isoleucine	104.80
**Vitamins**		L-Leucine	104.80
Choline Chloride	4.00	L-Lysine · HCl	146.20
Folic Acid	4.00	L-Methionine	30.00
myo-Inositol	7.00	L-Phenylalanine	66.00
Nicotinamide	4.00	L-Serine	42.00
D-Pantothenic Acid, Hemicalcium Salt	4.00	L-Threonine	95.20
Pyridoxal · HCl	4.00	L-Tryptophan	16.00
Riboflavin	0.40	L-Tyrosine · Na_2 · $2H_2O$	103.79
Thiamine · HCl	4.00	L-Valine	93.60
**Other components**			
D-Glucose	1000.00	Sodium Bicarbonate	3700.00

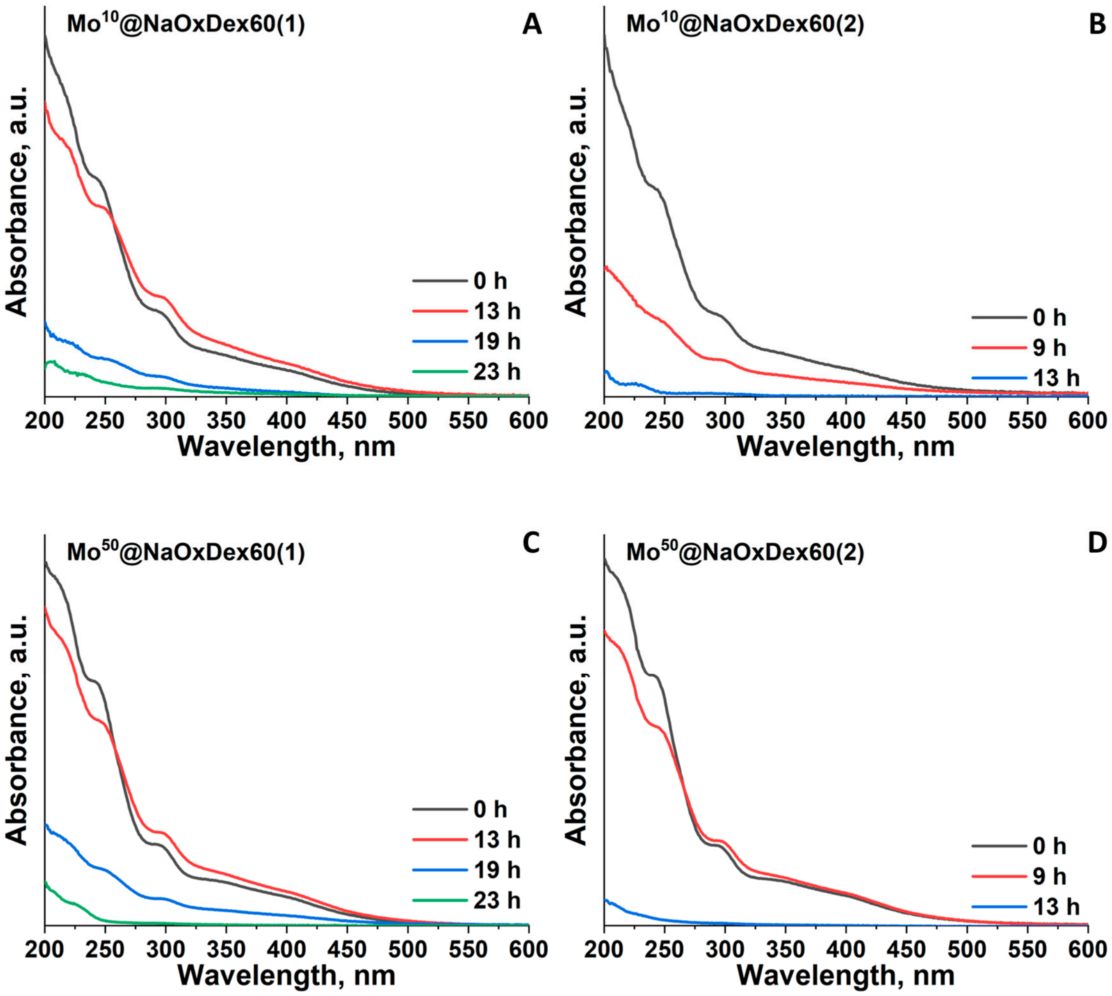

Figure S4. UV-vis spectra of Mo^x@NaOxDex60(n) (A – x = 10, n = 1; B – x = 10, n = 2; C – x = 50, n = 1; D – x = 50, n = 2) in DMEM culture medium over time.

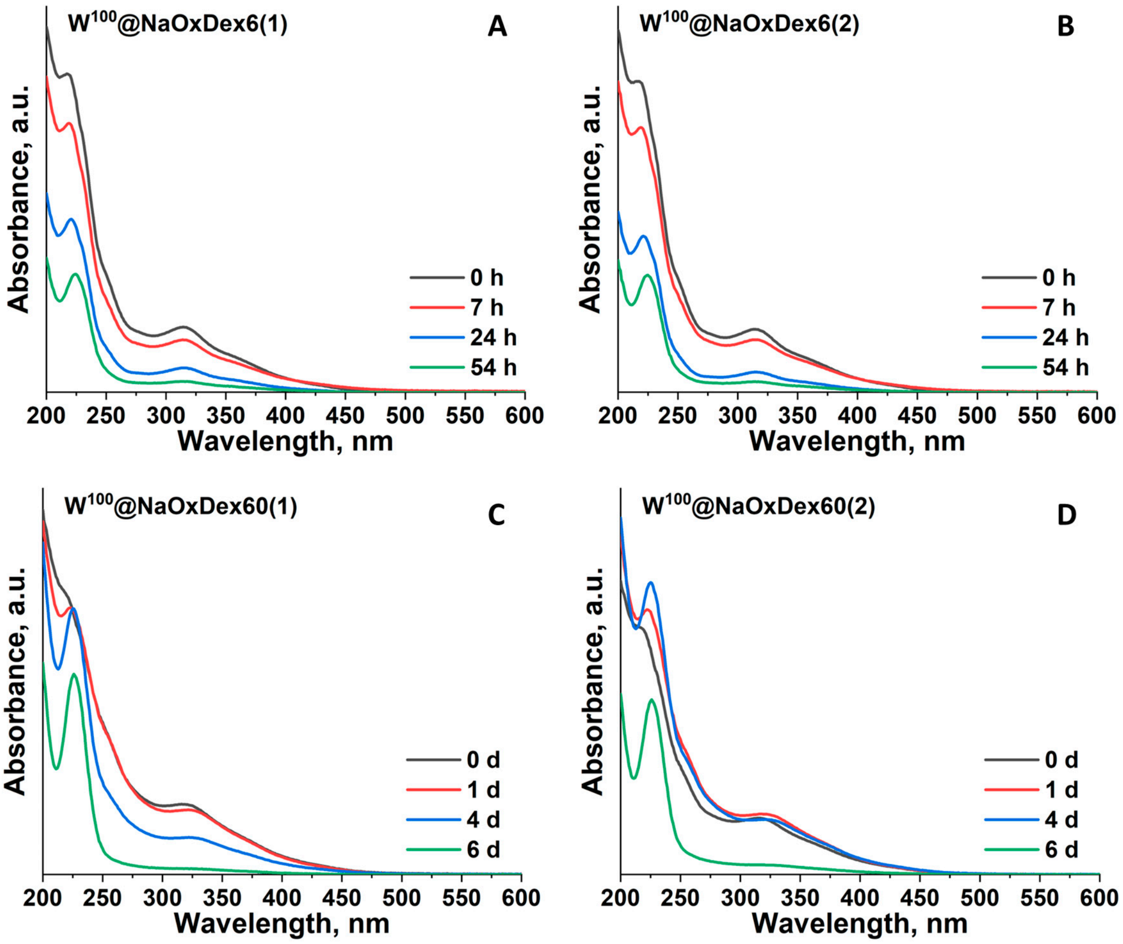

Figure S5. UV-vis spectra of W^{100}@NaOxDexQ(n) (A – Q = 6, n = 1; B – Q = 6, n = 2; C – Q = 60, n = 1; D – Q = 60, n = 2) in DMEM culture medium over time.

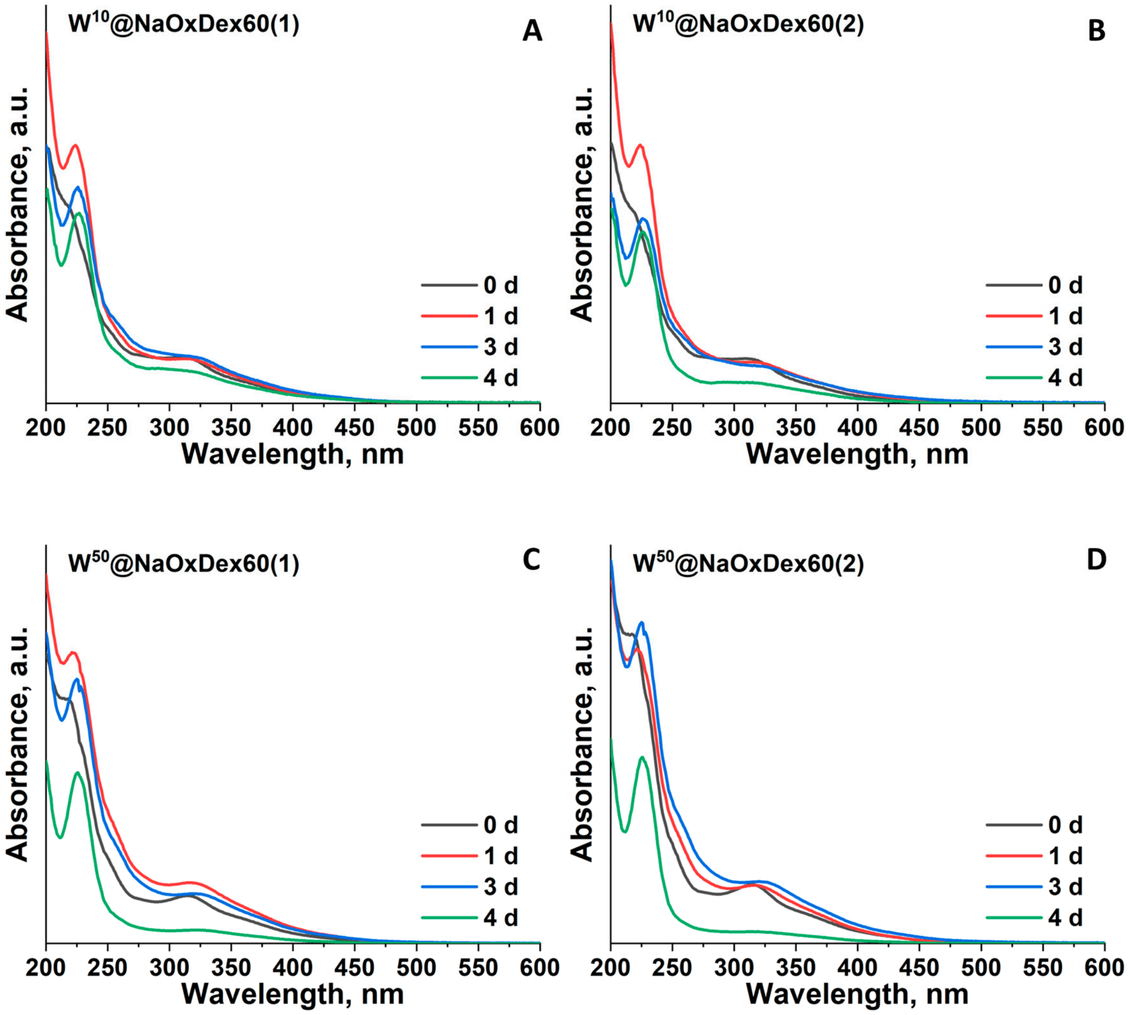

Figure S6. UV-vis spectra of W^x@NaOxDex60(n) (A – x = 10, n = 1; B – x = 10, n = 2; C – x = 50, n = 1; D – x = 50, n = 2) in DMEM culture medium over time.

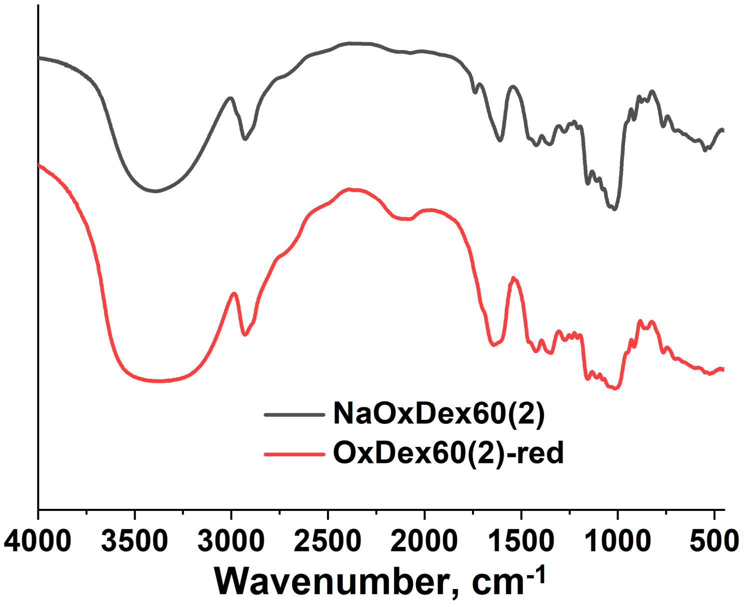

Figure S7. FTIR spectra of NaOxDex60(2) and NaOxDex60(2)-red.

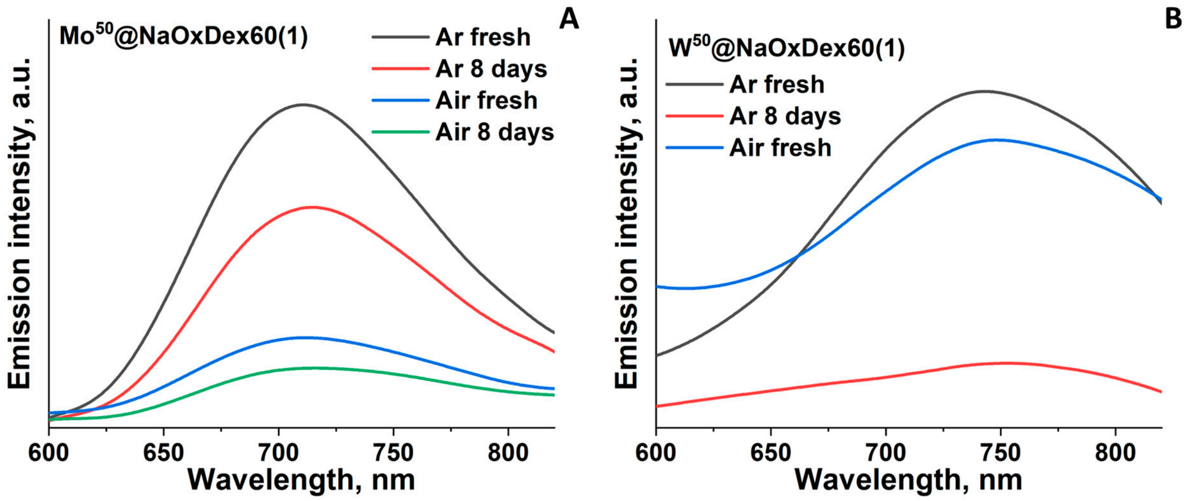

Figure S8. Emission spectra of fresh and 8-days old solution of Mo^{50}@NaOxDex60(1) in PBS (air and Ar) (A); Emission spectra of fresh and 8-days old solution of W^{50}@NaOxDex60(1) in PBS (air and Ar) (B).
